# Factors shaping community assemblages and species co‐occurrence of different trophic levels

**DOI:** 10.1002/ece3.3061

**Published:** 2017-05-23

**Authors:** Valeria Trivellone, Stephanie Bougeard, Simone Giavi, Patrik Krebs, Diego Balseiro, Stephane Dray, Marco Moretti

**Affiliations:** ^1^ Biodiversity and Conservation BiologySwiss Federal Research Institute WSLBirmensdorfSwitzerland; ^2^ Laboratory of Soil BiodiversityUniversity of NeuchâtelNeuchâtelSwitzerland; ^3^ Department of EpidemiologyAnses (French Agency for Food, Environmental and Occupational Health Safety)PloufraganFrance; ^4^ Community EcologySwiss Federal Research Institute WSLCadenazzoSwitzerland; ^5^ Centro de Investigaciones en Ciencias de la Tierra (CICTERRA)CONICET and Universidad Nacional de CórdobaCórdobaArgentina; ^6^ Laboratoire de Biométrie et Biologie EvolutiveUniv Lyon, Université Claude Bernard Lyon 1CNRSVilleurbanneFrance

**Keywords:** biotic and abiotic factors, leafhoppers, multiblock Redundancy Analysis, plants, trophic interactions, Variation partitioning

## Abstract

Species assemblages are the results of various processes, including dispersion and habitat filtering. Disentangling the effects of these different processes is challenging for statistical analysis, especially when biotic interactions should be considered. In this study, we used plants (producers) and leafhoppers (phytophagous) as model organisms, and we investigated the relative importance of abiotic versus biotic factors that shape community assemblages, and we infer on their biotic interactions by applying three‐step statistical analysis. We applied a novel statistical analysis, that is, multiblock Redundancy Analysis (mbRA, step 1) and showed that 51.8% and 54.1% of the overall variation in plant and leafhopper assemblages are, respectively, explained by the two multiblock models. The most important blocks of variables to explain the variations in plant and leafhopper assemblages were local topography and biotic factors. Variation partitioning analysis (step 2) showed that pure abiotic filtering and pure biotic processes were relatively less important than their combinations, suggesting that biotic relationships are strongly structured by abiotic conditions. Pairwise co‐occurrence analysis (step 3) on generalist leafhoppers and the most common plants identified 40 segregated species pairs (mainly between plant species) and 16 aggregated pairs (mainly between leafhopper species). Pairwise analysis on specialist leafhoppers and potential host plants clearly revealed aggregated patterns. Plant segregation suggests heterogeneous resource availability and competitive interactions, while leafhopper aggregation suggests host feeding differentiation at the local level, different feeding microhabitats on host plants, and similar environmental requirements of the species. Using the novel mbRA, we disentangle for the first time the relative importance of more than five distinct groups of variables shaping local species communities. We highlighted the important role of abiotic processes mediated by bottom‐up effects of plants on leafhopper communities. Our results revealed that in‐field structure diversification and trophic interactions are the main factors causing the co‐occurrence patterns observed.

## 1 INTRODUCTION

At a given point in space and time, the composition of species assemblage is the result of at least two processes that have been concurrently brought to completion: dispersion and habitat filtering. Dispersion enables individuals to spread through different habitats, while habitat filtering (both abiotic and biotic) permits populations to persist (e.g., Chase & Myers, 2011; Maire et al., 2012). Usually more than one ecological process is involved in determining species association patterns, and these processes may be concatenated to each other by a hierarchical perspective of ecological filters acting on different spatial scales (Hillebrand & Blenckner, 2002). Considering the species pool at regional level, niche‐based theories assume that abiotic filtering and biotic interactions mainly shape species richness and composition (Chase & Leibold, 2003; Diamond, 1975).

Multivariate statistical approaches (such as principal component analyses and redundancy analyses) are used to resolve ecological issues as niche differentiation and partitioning (Göthe, Angeler, Gottschalk, Lofgren, & Sandin, 2013; Janžekovi & Novak, 2012). Asymmetrical canonical ordination methods are applied for modeling a response variable *Y* (i.e., species communities observed) using a set of explanatory variables assembled in a data matrix X (i.e., usually abiotic factors), providing the proportion of the variation of the response data matrix Y that is accounted for by the explanatory matrix X. However, one of the pivotal ecological challenges at the moment is to determine how to statistically include in the analyses the contribution of biotic interactions, especially given the fact that observed species interaction matrices (who interacts with whom) are lacking (Ovaskainen, Abrego, Halme, & Dunson, 2016; Wisz et al., 2013). Even though surrogates for biotic relationships or reduced matrices for species interactions could be included as predictors of community composition, the issue on how properly depict the different kind of asymmetrical and symmetrical interactions and coping with large biotic interaction matrices in statistical models is still poorly explored.

In this study, we investigated the relative importance of abiotic and biotic factors that influence assemblage patterns. We specifically considered the major abiotic stressors and two taxa from two trophic levels: primary producers (plants) and phytophagous insects (Hemiptera Auchenorrhyncha; hereafter leafhoppers). We selected phytophagous insects and their host plants as suitable model organisms because comprise a significant proportion of overall terrestrial macrobiodiversity (Siemann, Tilman, & Haarstad, 1999; Strong, Lawton, & Southwood, 1984). Among the phytophagous insects, leafhoppers have been considered excellent model taxon to understand mechanisms affecting patterns of species diversification due to their high levels of host–plant specificity, limited dispersal, and high rates of local endemism (Biedermann, Achtziger, Nickel, & Stewart, 2005; Hamilton, 1997; Nickel, 2003).

In order to better understand the mechanisms underlying plant and leafhopper assemblages and their relationship with regard to the degree of feeding specialization, this study aims to answer the following specific questions: (i) What is the relative importance of biotic and abiotic factors on species assemblages of plants and herbivores? (ii) Does the response of both communities suggest an interaction effect between abiotic and biotic factors? (iii) What kind of biotic interaction can be inferred from the species that co‐occur at the local level?

We expect that abiotic factors and biotic interactions jointly define the community patterns observed at regional level, and that environmental factors affect both directly (via environmental filtering) and indirectly (via their effect on biotic interactions) the species assemblages of the studied communities. We also expect nonrandom co‐occurrence patterns for species pairs of both plants and leafhoppers. In particular, plant–plant (p‐p) species pairs should show more significant negative associations in the dataset due to competition for resources (Soussana & Lafarge, 1998). Leafhopper–leafhopper (l‐l) species pairs should show both positive and negative significant associations, the former due to the high potential for leafhopper feeding diversification in grasslands as postulated in Ross (1957) and Dietrich (1997), and the latter due to the effect of dominant species or different host preference. Plant–leafhopper (p‐l) species pairs should also show positive and negative significant associations, due to the direct interaction in the food web as well as plant‐mediated and physical‐factors‐mediated competition (Werner & Peacor, 2003). To test these hypotheses, we first applied a novel analytical approach, that is, the multiblock Redundancy Analysis (mbRA) to partition the effects of several groups of predictors expected to shape communities of plants and herbivores. We then investigated the extent of co‐variation between abiotic and biotic factors by means of Variation partitioning. Finally, we used co‐occurrence analysis to infer about the strength of potential biotic interactions based on patterns of co‐occurrence among species. The study was conducted in 48 vineyards distributed on both flat and terraced areas, offering a large environmental gradient across the region of Southern Switzerland (Trivellone et al., 2014).

## 2 MATERIALS AND METHODS

### 2.1 Study area

The investigation was conducted in a wine producing area south of the Swiss Alps, scattered over a region covering nearly 3,000 km^2^ and containing about 1,050 ha of vineyards. Forty‐eight vineyard fields were selected according to a stratified random selection process, which accounts for three abiotic factors (slope, aspect, and surrounding landscape) that affect biological communities in different ways. For a detailed description of the study area and field selection, see Trivellone et al. (2014).

### 2.2 Biological sampling

Plants and leafhoppers were sampled within three distinct homogeneous zones (hereafter sampling sites) within the vineyards: (i) ground row spacing (including grapevines with a standard width of 50 cm), (ii) flat ground inter‐row spacing between grapevine rows (width ranging from 155 to 185 cm), and (iii) ground slope inter‐row spacing (embankments). Both (ii) and (iii) are permanently covered by native plant communities (for details, see the scheme in Appendix S1). Overall, 68 sampling sites were considered in this study. Each site was sampled with different methods according to the taxon type.

Plants—surveys were conducted in two sampling periods (June and August, 2011) to account for early and late growing plant species. Percentage cover of vascular plant species was estimated in five 1 m × 1 m plots randomly distributed over each sampling site using a decimal scale after Londo (1976). Species nomenclature follows Lauber and Wagner (2009).

Leafhoppers—samplings were carried out in 2011 over eight periods at monthly intervals from March to October, covering the main activity period of leafhopper adults in vineyards. Based on a pilot survey (Trivellone, Paltrinieri, Jermini, & Moretti, 2012), four complementary sampling techniques were used in order to effectively capture most of the occurring species, and included the use of D‐vac suction sampler, beating tray, pitfall traps, and yellow sticky traps (for details, see Trivellone, Filippin, Narduzzi‐Wicht, & Angelini, 2016). All adults were identified to the species level by the first author and were preserved in 70% alcohol. Nomenclature followed Ribaut (1936, 1952), and Holzinger, Kammerlander, and Nickel (2003).

### 2.3 Response variables

The final dataset included 259 vascular plants and 166 leafhoppers species which were sampled in 68 sampling sites. Before analyses, the plant species cover percentage values were log‐transformed in order to reduce the influence of highly abundant and variable species. Leafhoppers abundance data were instead Hellinger‐transformed to reduce the influence of both extreme values and double absences in the data matrix (Legendre & Gallagher, 2001). All species occurring in less than five sites were removed from the analyses to avoid undesirable effects of both very rare species as well as vagrant and accidental individuals. The reduced dataset contained 117 vascular plants and 77 leafhoppers species.

All leafhopper species were classified in two major functional guilds based on diet breath of dietary specialization (Nickel & Remane, 2002): (a) specialists, species with very narrow food plant spectra, including monophagous and oligophagous species feeding on one or two species plant from a single genus, respectively; and (b) generalists, species with broader diets, that is, polyphagous species feeding on species plant from more than one genus.

### 2.4 Explanatory (abiotic and biotic) variables

We grouped the variables in seven thematic datasets (hereafter blocks), six blocks for abiotic, and one for biotic variables (for details, see Appendix S2). Block 1: Management (Man), consisting of five variables: mowing of vegetation, application of herbicides, fertilizers, insecticides, and fungicides. Block 2: Topography (Top), five variables: altitude, slope, aspect, solar radiation, and number of solar hours. Block 3: Chemical and physical properties of soil (Soil), nine variables: organic matter content, calcium carbonate, clay, sand, silt, total nitrogen, carbon/nitrogen ratio, inorganic nitrogen, and pH of soil. Block 4: Plant structure of cover vegetation (Struc), five variables: cover percentage of grass, moss, bare soil, rock, and litter. Block 5: Landscape composition within a 200‐m radius surrounding the sampled sites (Land200), six variables: covered area by vineyards, open vegetated areas, fellows, forests, settlements, and water bodies. Block 6: Landscape composition within a 500‐m radius (Land500), consisting of the same six variables as defined in Block 5. Block 7: biotic variables (Biotic), two variables defined as the first two components of a partial least‐squares regression analysis—PLSR (Wold, 1966) performed on the plant and leafhopper community matrices (for details, see *Statistical analyses* and Appendix S3).

### 2.5 Statistical analyses

**Figure 1:**
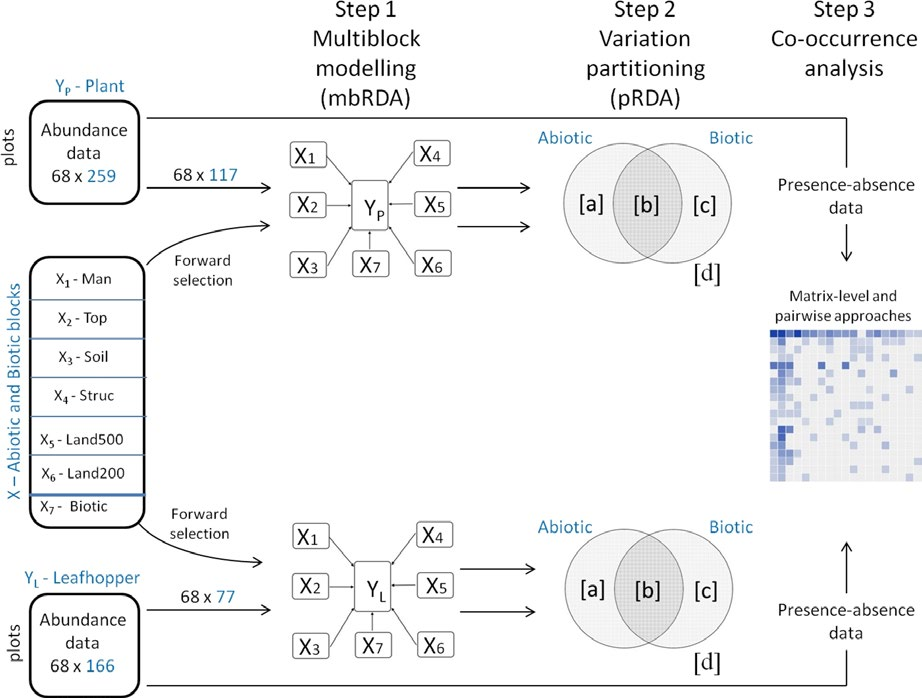
Overview of the statistical approach encompassing three steps. Step 1: X—Abiotic and biotic factors: X_1_—Man, management; X_2_—Top, topography; X_3_—Soil, chemical and physical property of soil; X_4_—Struc, structure of ground floor vegetation; X_5_—Land500, landscape composition defined within a 500‐m radius; X_6_—Land200, landscape composition defined within a 200‐m radius around the investigated vineyard; X_7_—Biotic, first two PLRS components of a Partial Least‐Squares Regression analysis. Step 2: the total variation of the dependent matrix was partitioned into four fractions: [a] pure abiotic factors; [b] a pure biotic factors; [c] shared variance; [d] unexplained variance

The response matrices and the seven explanatory matrices (i.e., blocks) were analyzed using a three‐step statistical approach (Figure 1). All analyses were performed using R (R Development Core Team 2010), unless otherwise specified.

*Step 1*—As traditional methods (such as Redundancy Analysis—RDA) do not adequately take into consideration the block structure of predictors and are not useable in this case due to the high number of possibly collinear explanatory variables, we proposed here a novel approach: the multiblock Redundancy Analysis (mbRA). We used mbRA to study variation in response variables (*Y*) that can be explained by K meaningful blocks of explanatory variables (*X*_*i*_) where *i *= 1, …, *K* (Bougeard, Qannari, & Rose, 2011). The two models are defined as follows:

(1) $$Y_{\mathrm{plant}}∼\text{Man}+\text{Top}+\text{Soil}+\text{Struc}+\text{Land200}+\text{Land500}+{\text{Biotic}}_{\mathrm{leafhopper}}$$

(2) $$Y_{\mathrm{leafhopper}}∼\text{Man}+\text{Top}+\text{Soil}+\text{Struc}+\text{Land200}+\text{Land500}+{\text{Biotic}}_{\mathrm{plant}}$$

where *Y*_plant_ and *Y*_leafhopper_ are the restricted datasets used as response variables in the multiblock models; Man, Top, Soil, Struc, Land200, and Land500 are the six blocks containing the abiotic predictors, and Biotic_leafhopper_ and Biotic_plant_ represent the biotic components resulting from the PLSR analysis.

The key idea behind this method is that each of the (*K* + 1) tables is summed up as a component, which is a linear combination of the raw variables. Using components instead of raw data enables more explanatory variables to be handled than in standard analyses, and restricts the problem of multicollinearity within explanatory blocks. This is the pivotal principle of orthogonalized regression, as components are not only the best summary of the raw data but also orthogonal with each other (Massy, 1965). More precisely, this method derives *K* block components, which are linear combinations of each block of explanatory variables, intended to be as close as possible a dependent linear combination of the response variables within each block. In addition, a global explanatory component related to all the explanatory variables is found as the best summary of the block components while taking into account their block structure. This global component is used in regression models to avoid integrating too many multicollinear variables.

As solutions are rarely unidimensional and in order to improve the prediction ability of the model, higher‐order solutions (i.e., dimensions) are obtained by considering the residuals of the orthogonal projections of the block data onto the subspace spanned by the first global component. From a practical point of view, the optimal regression model is obtained by selecting the optimal number of components to be introduced with a validation technique such as cross‐validation.

As in standard component analysis such as principal component analysis, the importance of each dimension is assessed by their inertia. In addition, the percentages of explained variances of each dataset Y and Xk by the global components are also an interesting information.

Besides the standard regression coefficients between explanatory and dependent variables, two useful indices are produced: (i) the Variable Importance index (VarImp), which enables the sorting of explanatory variables (*P*) by order of priority when the number of variables in *Y* is large, and (ii) the Block Importance index (BlockImp), which assesses the contributions of the explanatory blocks (*K*) to the overall dependent explanation. The detail for their interpretation is given in Appendix S3. For each abiotic block, only significant variables resulting from forward selection analyses (*P* = 0.05 after 9,999 random permutations) using Blanchet, Legendre, and Borcard (2008) double‐stopping procedure (to minimize the problems of the classical forward selection method) were included in the analysis. To run mbRA, we used the function *mbpcaiv* in the “ade4” R package (Dray & Dufour, 2007) combined with the function *forwards.sel* in the “packfor” R package for the forward selection (Dray, Legendre, & Blanchet, 2007).

*Step 2*—Variation partitioning was used to quantify the pure and shared contribution of abiotic and biotic factors in explaining the variation of plant and leafhopper communities at each sampling site (Anderson & Cribble, 1998; Borcard, Legendre, & Drapeau, 1992; Legendre & Legendre, 1998), as well as the portion of the variation explained by biotic factors structured by local abiotic conditions. Two matrices, that is, the abiotic matrix containing all significant abiotic variables combined and the biotic matrix with the first two PLRS components of plants and leafhoppers, respectively, were analyzed through a series of partial redundancy analyses (pRDA). The pRDA allows the total variation of response variables (plant or leafhopper community) to be partitioned into four fractions corresponding to pure abiotic, pure biotic, biotic variance structured in the abiotic fraction (shared fraction), and unexplained variation (Borcard, Gillet, & Legendre, 2011; Peres‐Neto, Legendre, Dray, & Borcard, 2006). The variation explained of each fraction was reported as the adjusted coefficient of multiple determination (*R* ^2^_adj_) to take into account the number of explanatory variables and sample size while preventing the inflation of *R* ^2^ values (Peres‐Neto et al., 2006). The significance of each source of variation was tested with a Monte Carlo permutation test (999 permutations). The analyses were performed with the *varpart* function in the “vegan” R package.

*Step 3*—The species co‐occurrence analysis was performed using two different approaches: (i) matrix‐level, and (ii) pairwise (Gotelli, 2000; Veech, 2014). In both cases, we used a presence–absence community matrix from the final plant and leafhopper datasets, respectively.

The *matrix‐level approach* was applied to plant and leafhopper datasets separately with the aim to describe the overall patterns of species occurrences. The null hypothesis is that replicated local assemblages are not significantly different from those expected by chance. The rejection of the null hypothesis indicates that the underlying mechanisms acting on species assemblages may reflect species interaction, abiotic filtering, or dispersal limitation. To assess the co‐occurrence patterns, we used the fixed–fixed (FF) algorithm for randomization and the C‐score index to measure the degree of segregation across sampling sites (for further details see Appendix S3).

The *pairwise approach* was used to identify which species pairs co‐occurred more or less frequently than expected by chance, and whether leafhopper feeding specialization (specialists versus generalists) showed different patterns. Two submatrices of plant–leafhoppers were analyzed to identify the observed patterns: a submatrix of 150 rows (62 Generalist leafhoppers and the 88 most widespread and abundant plants in the study sites—Common plants) by 68 sampling sites (hereafter matrix G‐C), and a submatrix of 90 rows (56 Specialist leafhoppers and 34 potential Host plants) by 68 sampling sites (hereafter matrix S‐H). In the S‐H matrix, those leafhopper species with specialized feeding behavior and their plant hosts were selected according to the literature (Nickel & Remane, 2002). If none were recorded in the survey, a congenus species was selected. To test the nonrandom patterns of co‐occurrence, we used the FF algorithm. The C‐score for each species pair was calculated, and the significance was determined using confidence limits based on the random distributions (standard contour length, hereafter CL criterion). As this method is potentially prone to large Type I errors, the more restrictive empirical Mean Bayes (Bayes M) method was also applied (Gotelli & Ulrich, 2010). The R package “EcoSimR” was used for the matrix‐level approach to the species co‐occurrence analyses, while the pairwise co‐occurrence analyses were performed using the PAIRS program (Ulrich, 2008).

## 3 RESULTS

### 3.1 Factors driving plant communities (Step 1)

Among the 36 abiotic variables, 16 were significant for the plant community matrix based on the forward selection analyses (Appendix S2) and were selected for *Step 1*. The first two PLSR components of the leafhopper communities explained 31.2% and 13.2% of the variance of the plant communities, respectively. The first two dimensions of mbRA explained 32.1% of the total inertia (respectively, 23.4% and 8.7%), 30.2% of the plant community matrix variance and 41.0% of the predictor variance (for details, see Appendix S4).

The optimal model for plant communities (Equation (1)) was obtained by selecting components after a twofold cross‐validation. This model explains 51.8% of the variation in plant communities, 60.2% in management (Man), 62.1% in topography (Top), 63.8% in chemical and physical properties of soil (Soil), 48.2% in plant structure of cover vegetation (Struc), 54.5% in landscape composition within a 200‐m radius (Land500), 70.5% in Landscape composition within a 500‐m (Land200), and 88.1% in the first two PLSR components (Biotic).

**Figure 2:**
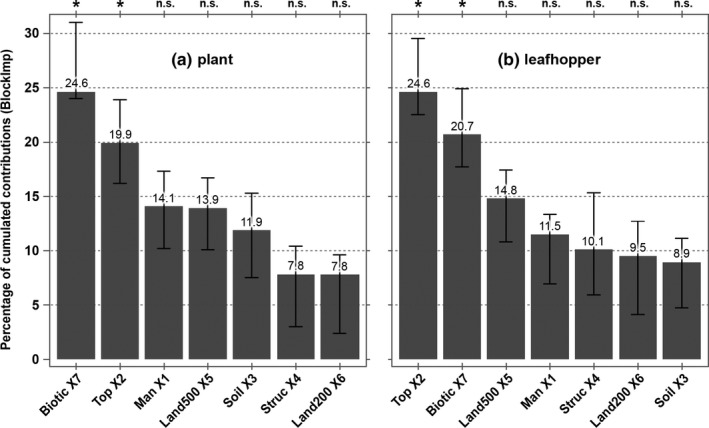
Multiblock modeling for plant (a) and leafhopper (b) communities—percentage of cumulated contributions (BlockImp) of each explanatory block (from X1 to X7) in the community prediction. The optimal model of mbRA involves *h* = 5 components. Significant blocks (*) are: X2 (topography) and X7 (PLRS components). Error bars indicate the 95% tolerance intervals. For abbreviations of block labels, see Figure 1

The Block Importance (BlockImp) index quantifies the contribution of the seven explanatory blocks (*K* from 1 to 7) in explaining plant community variation. The threshold value for block significance is set to 1/*K *= 0.14 (14.0%). Figure 2a shows the weighted cumulated indices over several components included in the model for each block. Plant communities are mainly driven by topographic (BlockImp = 19.9% [min: 16.2; max: 23.9]_95%_) and biotic (BlockImp = 24.6% [24.0; 31.0]_95%_) attributes.

**Figure 3:**
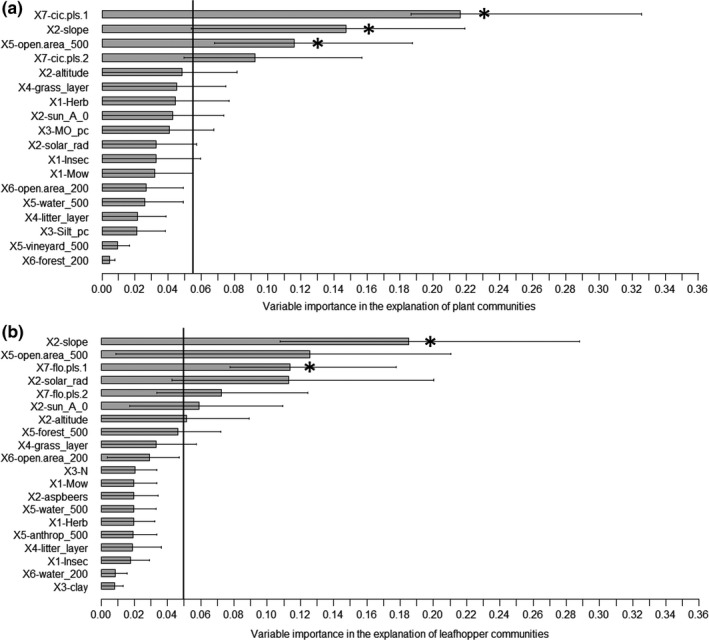
Contribution of the 18 explanatory variables to plant community variation (a) and of the 20 explanatory variables to leafhopper community variation (b), based on the Variable Importance index, with 95% tolerance intervals for the model involving five components. The vertical line is the threshold value (1/*P *= 0.055 for plant and 1/*P *= 0.05 for leafhopper communities), where *P* is the total number of variables in the model. Significant variables (*) for plant communities are: X7‐cic.pls.1 (the first leafhopper‐community PLSR component), X2‐slope (slope of sampling sites), and X5‐open area_500 (open area surrounding the vineyard within a 500‐m radius). Significant variables (*) for leafhopper communities are: X2‐slope (slope of area) and X7‐flo.pls.1 (the first plant‐community PLSR component)

Figure 3a shows the importance of the individual abiotic and biotic explanatory variables (*P*) on the plant community prediction calculated by means of the Variable Importance (VarImp) index with associated standard deviations and tolerance intervals. It enables the sorting of the *P *= 18 abiotic and biotic variables by overall order of priority. The threshold value for variable significance is set to 1/*P *= 0.055 (5.5%). Of 18, three significant variables affecting plant communities were identified: the first leafhopper‐community PLSR component (VarImp = 21.6% [18.7; 32.6]_95%_, X_7_), the slope of sampling sites (VarImp = 14.7% [min: 5.4; max: 21.9]_95%_, block: X_2_), and the open area surrounding the vineyard within a 500‐m radius (VarImp = 11.6% [6.8; 18.8]_95%_, X_5_).

### 3.2 Covariation between abiotic and biotic variables for plant communities (Step 2)

**Figure 4:**
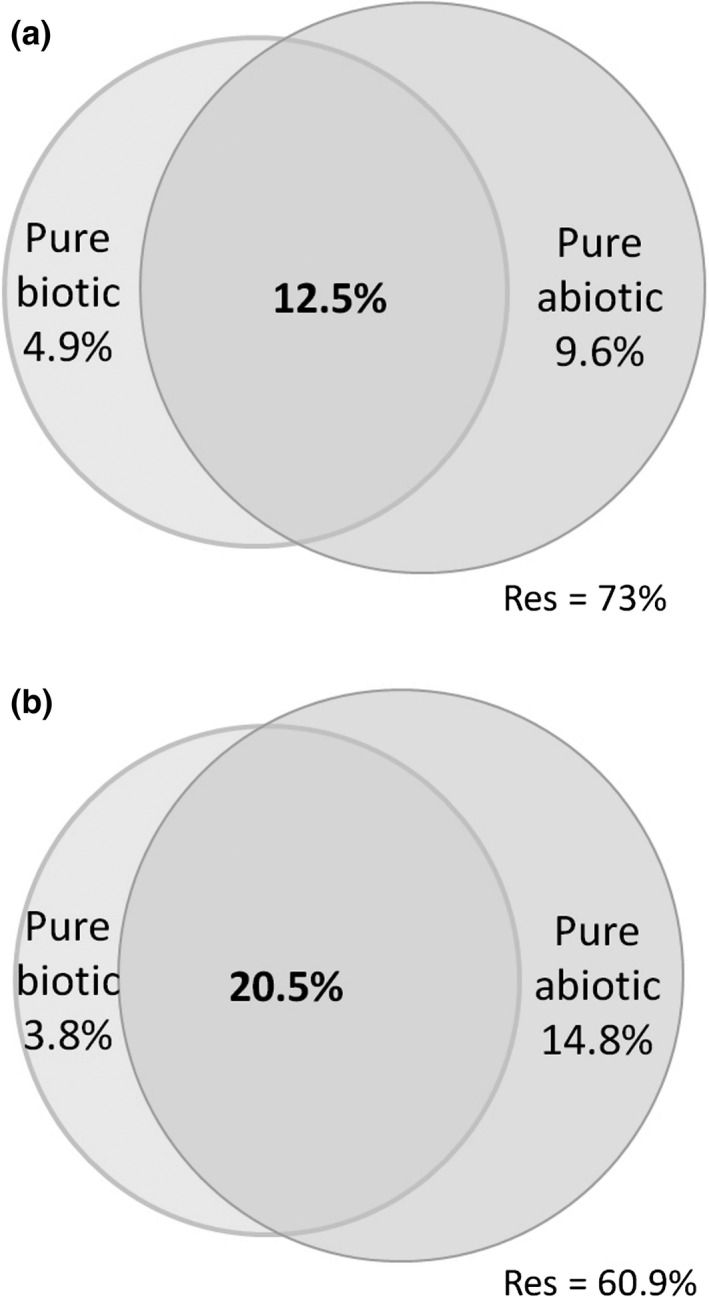
Variation partitioning of plant (a) and leafhopper (b) communities in each sampling site tested by partial redundancy analyses (pRDA) with the percentage of variance explained (*R* ^2^_adj_) by the pure biotic fraction, pure abiotic fraction, the shared fraction, and the unexplained fraction (Res)

The amount of variation explained by the abiotic‐biotic shared fraction (*R* ^2^_adj_ = 12.5%) was higher than pure abiotic (*R* ^2^_adj_ = 9.6%) and biotic (*R* ^2^_adj_ = 4.9%) contributions. This overlap indicates that biotic factors are mainly structured by abiotic characteristics (Figure 4a).

### 3.3 Factors driving leafhopper communities (Step 1)

Among the 36 abiotic variables, 18 significantly explained leafhopper species assemblages after the forward selection analyses (Appendix S2) and were selected for *Step 1*. The first two plant‐community PLSR components explained 30.1% and 13.1% of the variance in the response variable (leafhopper communities), respectively.

The first two dimensions of mbRA explained 34.1% of the total inertia (respectively, 22.3% and 11.7%), 31.2% of the leafhopper community matrix variance, and 39.8% of the predictor variance (for details see Appendix S4).

The optimal model for leafhopper communities (Equation (2)) was obtained by selecting five components after a twofold cross‐validation. This model explains 54.1% of the variation in *Y*, 54.4.2% in Man, 60.4% in Top, 57.5% in Soil, 70.2% in Struc, 61.2% in Land500, 62.8% in Land200, and 84.2% in Biotic.

The Block Importance (BlockImp) index quantifies the contribution of the seven explanatory blocks in explaining leafhopper community variation. The threshold value for the block significance is set to 1/*K *= 0.14 (14.0%). Figure 2b shows the weighted cumulated indices over several components included in the model for each block. Overall, leafhopper communities are driven by topographic (BlockImp = 24.6% [min: 22.5; max: 29.5]_95%_) and biotic (BlockImp = 20.7% [17.7; 24.9]_95%_) attributes.

Figure 3b shows the importance of the individual abiotic and biotic explanatory variables (*P*) on the leafhopper community prediction calculated by means of the Variable Importance (VarImp) index with associated standard deviations and tolerance intervals. It enables the sorting the *P *= 20 abiotic and biotic variables by overall order of priority. The threshold value for variable significance is set to 1/*P *= 0.05 (5.0%). Of 20, two significant variables affecting leafhopper communities were identified: the slope of the area (VarImp = 18.5% [10.8; 28.8]_95%_, X_2_) and the first plant‐community PLSR component (VarImp = 11.4% [7.8; 17.8]_95%_, X_7_).

### 3.4 Covariation between abiotic and biotic variables for leafhopper communities (Step 2)

The amount of variation accounted for by the abiotic‐biotic shared fraction (*R* ^2^_adj_ = 20.5%) was higher than pure abiotic (*R* ^2^_adj_ = 14.8%) and biotic (*R* ^2^_adj_ = 3.8%) contributions. This overlap indicates that biotic factors are mainly structured by abiotic characteristics (Figure 4b).

### 3.5 Species co‐occurrence (Step 3)

The results from the null model using the matrix‐level approach on plant and leafhopper presence–absence matrices indicate that species co‐occurrence is not random in our datasets. In the plant community matrix, the C‐score index was significantly larger than that expected by chance (*p*‐value <.001), indicating nonrandom species segregation; in the leafhopper community matrix, the same trend was observed (*p*‐value <.001).

The pair co‐occurrence analysis on the G–C matrix (i.e., Generalist leafhoppers and the most Common plants) identified a total of 380 significant unique species pairs based on the CL criterion. Of these 380 pairs, 56 were significant based on the Mean Bayes criterion (Appendix S5). C‐score indices identified 40 segregated species pairs (five leafhopper–leafhopper “l‐l”; 12 plant–plant “p‐p” and 23 “p‐l”) and 16 aggregated species pairs (14 “l‐l”; 0 “p‐p” and two “p‐l”).

The pair co‐occurrence analysis on the S–H matrix (i.e., Specialist leafhoppers and potential Host plants) identified a total of 133 significant unique species pairs by CL criterion, 31 of which were also significant based on the Mean Bayes criterion (Appendix S6). All of them are aggregated species pairs (nine “l‐l”; seven “p‐p”; and 15 “l‐p”).

An overview of the main results of the application of the three‐step statistical framework is reported in Appendix S7.

## 4 DISCUSSION

Biotic and abiotic constraints are widely assumed to act together in accounting for the distribution of species and their abundances. Nonetheless, many studies usually focus on the effect of abiotic factors alone. Several statistical approaches have been developed to determine the relative contributions of different explanatory variables in their shaping of biological communities, but none of these has received full consensus inside the scientific community thus far (Wisz et al., 2013). In order to consider all major factors that affect plant and leafhopper communities, we used two different statistical approaches which provide complementary information: multiblock modeling (mbRA) and Variation partitioning. The mbRA approach was originally developed for epidemiological analysis (Bougeard & Cardinal, 2014; Bougeard, Lupo, Le Bouquin, Chauvin, & Qannari, 2012; Bougeard et al., 2011). To our knowledge, the present study represents the first time that this method has been used in ecological research. This method allows for the assessment of the influence of more than four groups of explanatory variables on community assemblages taking into account both the contribution of group of variables (blocks) and single variables. Variation partitioning is uniquely suited to estimate the shared variation among groups of explanatory variables (maximum four groups). By means of co‐occurrence analyses, we were able to make hypothesis on the possible interactions shaping coexistence between species. While observational studies such as the present investigation may limit the assessment of underlying mechanisms as pointed out by Kraft et al. (2015), the combination of methods used here nevertheless permits a better understanding of the forces that drive community assemblages than either of them used separately.

### 4.1 Relative importance of biotic and abiotic variables

Our study region is characterized by strong environmental gradients acting at different scales and quite a heterogeneous land morphology and diversified landscape. Accordingly, our data show that more than half of the variation across plant (51.8%) and leafhopper (54.1%) assemblages is mainly driven by habitat filtering processes, in particular topography (slope of study site and open green areas within a 500‐m radius) and, to a lesser degree, by biotic factors mainly structured by habitat conditions. In addition, the contribution of pure abiotic factors was higher compared to pure biotic factors in both plants (9.6% vs. 4.9%) and leafhoppers (14.8% vs. 3.8%), respectively, indicating that abiotic filtering processes are relatively more important than biotic factors in shaping community assemblages at both trophic levels. Steep slopes appear to represent an important component of vineyards in mountain and hilly regions in Switzerland, which create an in‐field differentiation by means of zones inside vineyards (inter‐row, row, and embankment), in the same way that green open areas in neighboring vineyards create different patches of plant communities (meadows, grasslands, and fellows), which may serve as areas for plant dispersal. Microhabitat differentiation inside vineyards and patches in their surroundings create a mosaic of environments, which improves the conditions for colonization of different assemblages of plants with different environmental requirements (abiotic filtering). The variation on leafhopper communities driven by pure abiotic factors (slope of sites) conceals the influence due to the plant community variability, which influences the availability and quality of food for leafhoppers. Our findings are consistent with the results of Sanderson, Rushton, Cherrill, and Byrne (1995), who showed that leafhopper assemblages are primarily affected by vegetation species composition and structure. It should be noted, however, that other factors can indicate a mediation effect, as highlighted by Kőrösi, Batáry, Orosz, Rédei, and Báldi (2012) in their study of semi‐natural grasslands in Hungary. This study, partly consistent with our results, revealed that factors such as management, vegetation structure, and landscape act strongly together in affecting leafhopper assemblages. The results suggest that the variability of plant and leafhopper assemblages is due to structural factors within the vineyards and composition of surrounding landscape. On the other hand, the variation in plant and leafhopper communities is to a greater degree explained by the overlap (12.5% and 20.5%, respectively) between abiotic and biotic variables, which account for significant amounts of variance. Thus, shared variance could represent biotic relationships that are influenced by abiotic factors, or the joint effect of abiotic factors structuring the biotic component of a different trophic level. Our hypothesis is that leafhopper and plant communities are structured by the steep environmental gradient created by the slopes of sampling sites and the surrounding landscape. A similar trend was observed by Göthe et al. (2013) on diatoms and invertebrate grazers in Swedish headwater streams where a high proportion of the variance was explained by overlap between abiotic and biotic factors. The authors suggested that the pronounced environmental gradient in the dataset may have overridden trophic interactions. Similar evidence is also found in our study based on the results of the mbRA (variable importance of biotic relationship factors).

Although the pure biotic contribution emerging from pRDA for both leafhopper and plant communities is lower, in our opinion it is due to a bottom‐up effect. This is consistent with previous findings for other similar terrestrial ecosystems. For example, Rzanny, Kuu, and Voigt (2013) support the view that plants predominantly determine consumer community composition of various trophic levels.

We hypothesize that the relatively small fraction of pure biotic factors affecting the communities of plants (4.9%) and leafhoppers (3.8%), respectively, could reflect actual biotic interactions between plants and leafhoppers as shown by the pairwise co‐occurrence analyses.

### 4.2 Groups of co‐occurring species pairs

Null model tests have been successfully applied to terrestrial animal communities with the aim of investigating co‐occurrence patterns (Gotelli & Ellison, 2002; Ingimarsdóttir et al., 2012; Jiménez, Decaëns, & Rossi, 2012; Lin et al., 2014). Species co‐occurrence using the matrix‐level approach for plant and leafhopper assemblages in the present study show clear evidence of nonrandomness patterns where species assemblages mainly segregate, as indicated by the co‐occurrence rate found that is significantly lower than that expected by chance. As highlighted by Gotelli and McCabe (2002), many empirical datasets exhibit segregation patterns, even if they necessarily contain some species pairs that are aggregated (Stone & Roberts, 1990). Many studies have revealed that overall segregated patterns do not necessarily suggest competition, but could emerge as a result of differentiation in habitat requirements or phylogenetic history (Ulrich & Gotelli, 2013). Usually, these processes are never mutually exclusive (Ricklefs & Schluter, 1993). Ulrich and Gotelli (2012) pointed out the advantages of using a fixed–fixed (FF) algorithm, which respects the relative contribution of factors that are not related to species interactions which may influence widespread heterogeneity in species richness and species occurrences. As plant and leafhopper assemblages were also significantly affected by the topographic characteristics of the sampling sites (see results from multiblock Redundancy Analyses), choosing a FF algorithm was the best choice. The results of pairwise co‐occurrence analyses on the Generalist leafhoppers‐Common plant species (G–C) matrix shows a high frequency of segregated species pairs (40 of 57), which is consistent with the co‐occurrence pattern of entire communities. Among them, the segregated pattern was more important within plant communities where all selected species pairs (12) were segregated, and just in few cases pairs showed contrasting environmental requirements (e.g., *Veronica arvensis* Linnaeus occurred usually on dry soils and *Rumex acetosa* Linnaeus mainly in wet meadows). The observed plant segregated patterns can reasonably be attributed here to the net effect of abiotic filtering (Pulliam, 2000), heterogeneous resource availability and competitive interactions (Diamond, 1975; Gotelli, 2000), leading to niche differentiation among species, which is also consistent with widespread evidence reported in Tilman (1982) and Keddy (1989). In contrast, leafhopper–leafhopper species pairs moved toward an aggregated pattern with 15 species pairs of 20 pairs in total. These results suggest that most polyphagous and common leafhoppers co‐exist in vineyard agroecosystems. This likely occurs because host feeding differentiation at the local level, different feeding microhabitats on host plants and similar environmental requirements (Sanders et al., 2007) between species could result in attraction even in the absence of interactions (e.g., facilitation; Novotny et al., 2012). For instance, *Laodelphax striatella* (Fallén) is aggregated with 12 different leafhopper species, all of which belong to different families or subfamilies and have different feeding sites on the host plant.

In our study, the overall observed segregated patterns of leafhoppers can be reasonably attributed mainly to abiotic factors (i.e., slope of sampling sites) mediated by a bottom‐up effect due to plant species diversity, as also shown in the first two steps of our analyses. Our findings suggest a potentially high leafhopper community diversification due to their strong ability to partition resources within the host plant (i.e., feeding site differentiation).

The pairwise co‐occurrence analyses of the Specialist leafhopper‐potential Host plant (S‐H) matrix clearly reveal an aggregated pattern. As expected, the majority of species pairs were selected from leafhopper‐plant associations, and species‐specific phytophagous‐host plant relationships were highlighted. Of 15 leafhopper‐plant species pairs, only one pair, *Horvathianella palliceps* (Horvath) and *Chrysopogon gryllus* (Linnaeus), confirms the relationship already reported in the literature, whereas another species pair, *Kelisia guttulifera* (Kirschbaum) and *Carex hirta* Linnaeus, confirms previous records at the genus level only (Nickel & Remane, 2002).

## 5 CONCLUSION AND PERSPECTIVES

Distinguishing between pure abiotic filtering and biotic interaction for the purpose of understanding the processes involved remains a challenge (Kraft et al., 2015). Our results reveal that plant and leafhopper communities are strongly driven by abiotic factors, which affect biotic relationships, as implied by the fact that the two linked trophic levels are mainly structured by bottom‐up forces. Moreover, our results provide evidence of co‐occurrence patterns established in both observed guilds, which, in turn, reveal that in‐field diversification and trophic interactions are the main factors which can cause segregation or aggregation of species.
