# Supplementary material for: Factors shaping community assemblages and species co‐occurrence of different trophic levels
Source: Ecol Evol. 2017 May 23;7(13):4745–54. doi: 10.1002/ece3.3061 (PMC5496552; doi:10.1002/ece3.3061)

Appendix S1.

Representation of a "vineyard model" partitioned in 3 homogeneous zones. **R** the ground row spacing (including grapevines with a standard width of 50 cm); **I** the flat ground inter-row spacing between grapevine rows (width ranging from 155 to 185 cm); and **S** the ground slope inter-row spacing (embankments). Both **I** and **S** are permanently covered by wild vegetation.

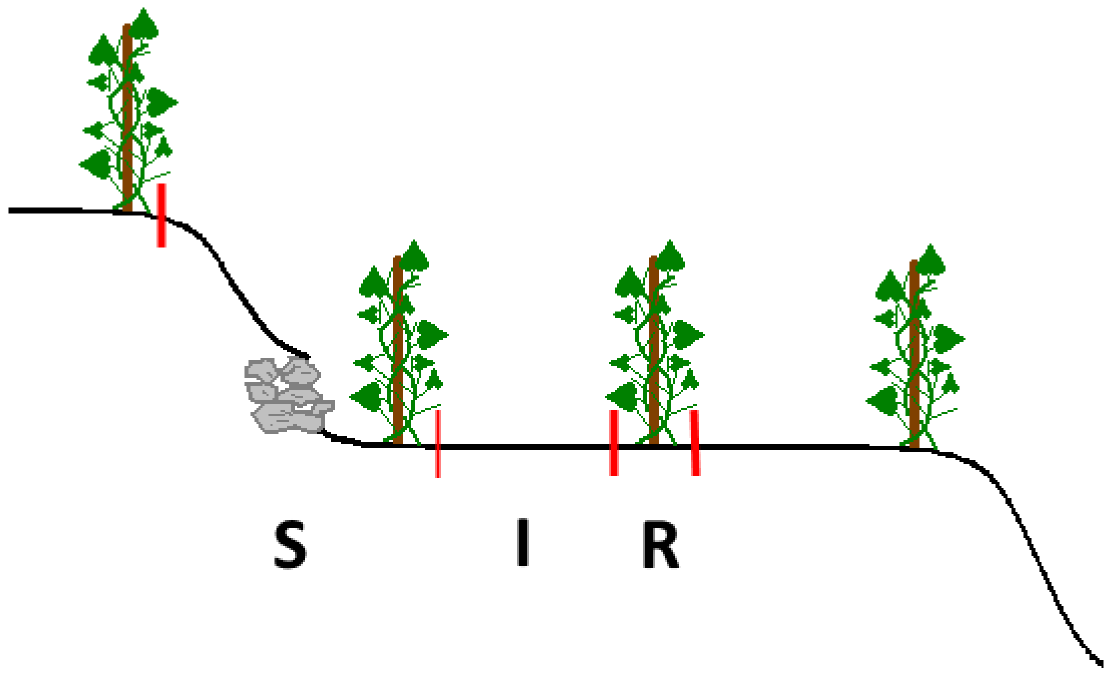

Supplement: Supplementary file 1 [file ECE3-7-4745-s001.pdf]
