# Supplementary material for: Factors shaping community assemblages and species co‐occurrence of different trophic levels
Source: Ecol Evol. 2017 May 23;7(13):4745–54. doi: 10.1002/ece3.3061 (PMC5496552; doi:10.1002/ece3.3061)
Supplement: Supplementary file 2 [file ECE3-7-4745-s002.pdf]

Appendix S2.

Overview of abiotic and biotic variables assigned to each investigated vineyard in 2011. (a) Significant abiotic variables after the forward selection procedure are reported for plant and leafhopper communities. (b) Management variables were collected by administering specific questionnaires to vinegrowers. (c) All physical and chemical analyses were carried out by SolConseil (Changins). (d) Vegetation structure were measured in August. A rectangular of about 800 x 250 m was placed around each pitfall trap station and percentage of each category was recorded. (e) Six landscape cover units have been defined and percentage of each unit was collected by means of georeferenced images and digital cartographic model of Switzerland (resolution of 25 x 25 m, Vector 25 of Swisstopo) based on the topographic 1:25,000 maps, using a geographic information system (ArcGis 10). Data were collected inside a circle of 500 m and 200m of radius around the investigated vineyard.

BLOCK		Unit	Significant variable (a) plant	Significant variable (a) leafhopper	Description
Abiotic variables					
[MAN] - Management (b)	Mowing	[n°/year]	Yes	Yes	Number of mowing of ground cover vegetation
	Herbicide	[n°/year]	Yes	Yes	Number of applications of herbicide (organophosphorus compounds and glufosinate, mainly)
	Fertilisers	[n°/year]	-	-	Number of applications of fertiliser (NPK: nitrogen, phosphorus and potassium)
	Insecticide	[n°/year]	Yes	Yes	Number of applications of insecticide (inhibitors of chitin synthesis)
	Fungicide	[n°/year]	-	-	Number of applications of fungicide (different formulations against Powdery Mildew, Downy Mildew and Black Rot)
[TOP] - Topography	Aspect	-	-	Yes	Xtr =cos(radianti(X-45°))+1 where X = number degrees
	Slope	[°]	Yes	Yes	Slope of vineyard and of each zone inside
	Solar Radiation	[W/m^2]	Yes	Yes	Mean of solar radiation in vegetative period (April-October) in 2011
	Solar time	[hours/day]	Yes	Yes	Number of sunlight hours measured on the center of vineyard
	Altitude	[m]	Yes	Yes	Altitude above see level
[SOIL] - Chemical and physical property (c)	Hand texture analysis	%	Silt	Clay	Proportion of: clay, silt, and sand
	MO	%	Yes	-	Organic matter content
	CaCO3	%	-	-	Total content of calcium carbonate

BLOCK		Unit	Significant variable (a) plant	leafhopper	Description
	pH	-	-	-	pH of two samples of soil
	Ntot	%	-	Yes	Total nitrogen content
	C/N	-	-	-	Carbon/nitrogen ratio
	N_{inorg}	[kg/ha]	-	-	Total inorganic nitrogen content
[STRUC] - Structure of ground vegetation	Grass	%	Yes	Yes	Categories of vegetation (d)
	Moss	%	-	-	
	Bare soil	%	-	-	
	Rock	%	-	-	
	Litter	%	Yes	Yes	
[LAND500] - Landscape	Vineyard	[m^2]	Yes	-	Landscape composition based on 6 cover units (e)
	Open area	[m^2]	Yes	Yes	
	Fallow	[m^2]	-	-	
	Forest	[m^2]	-	Yes	
	Settlement	[m^2]	-	Yes	
	Water	[m^2]	Yes	Yes	
[LAND200]- Landscape	Vineyard	[m^2]	-	-	Landscape composition based on 6 cover units (e)
	Open area	[m^2]	Yes	Yes	
	Fallow	[m^2]	-	-	
	Forest	[m^2]	Yes	-	
	Settlement	[m^2]	-	-	
	Water	[m^2]	-	Yes	
Biotic variables					
[BIOTIC $_{leafhopper}$] - Biotic contribution	Biotic relationship	-	-	-	first two components (or *latent vectors*) of partial least-squares regression analysis(PLRS), obtained from plant as response variable

**BLOCK**		**Unit**	**Significant variable (a)**		**Description**
			**plant**	**leafhopper**	
					and leafhopper communities as explanatory variables.
[BIOTIC $_{plant}$] - Biotic contribution	Biotic relationship	-	-	-	first two components (or *latent vectors*) of partial least-squares regression analysis (PLRS), obtained from leafhopper as response variable and plant communities as explanatory variables.
