# Supplementary material for: Factors shaping community assemblages and species co‐occurrence of different trophic levels
Source: Ecol Evol. 2017 May 23;7(13):4745–54. doi: 10.1002/ece3.3061 (PMC5496552; doi:10.1002/ece3.3061)
Supplement: Supplementary file 3 [file ECE3-7-4745-s003.pdf]

Appendix S3.

Details on statistical analyses used.

Step 1 - Partial Least-Squares Regression analysis (PLSR)

Concerning the biotic components ($\text{Biotic}_{\text{leafhopper}}$ and $\text{Biotic}_{\text{plant}}$) used in the multiblock models, our aim was to obtain a dimensionally reduced biotic matrix of independent predictor components which represents the biotic relationships between the two trophic levels (plant and leafhopper communities). To achieve this, we applied PLSR analysis rather than a non-constrained dimensional reduction technique (e.g. Principal Component Analysis), because the data values of both independent and dependent variables influence the construction of the PLSR components used as biotic blocks. For the $\textit{Biotic}_{\text{leafhopper}}$ block, the PLSR model will find the optimal and orthogonal directions in the leafhopper community space that explain the maximum multidimensional variability in the plant space; and vice versa for the $\textit{Biotic}_{\text{plant}}$ block. This analysis reduces the number of explanatory variables—which are similar to the to the number of response variables—and avoids the problem of correlated predictors (Carrascal, Galván & Gordo 2009). The first two vectors of PLSR components are considered for each biotic matrix. The analyses were performed using the R package 'pls'.

Multiblock Redundancy Analysis (mbRA) – Details for the interpretation of the multiblock indices

Both the indices, i.e., VarImp and BlockImp, measure the relative importance of the variables and the blocks, respectively, on the whole dependent block explanation. Indeed, as the sum of both these indices are constrained to be equal to one for all the explanatory variables and all the explanatory blocks, they actually assess the relative importance of the explanatory variables and blocks, to the overall dependent explanation. Associated standard deviations and tolerance intervals, computed using bootstrapped results may be given. As both these indices verify the property that their sum is equal to one, the threshold value of 1/K, resp. 1/P may be used where K is the number

of blocks and P the number of explanatory variables. It follows that a block is considered to be significantly associated with the dataset to be explained, if its 95% tolerance interval associated with the BlockImp, resp. VarImp, value does not contain the threshold value 1/K, resp. 1/P. See (Bougeard, Qannari & Rose 2011) for all the details about these multiblock indexes.

Step 3 - Species co-occurrence analysis

Matrix-level approach was used to describe patterns of species occurrences, where the null hypothesis is that replicated local assemblages are not significantly different from those expected by chance. The rejection of the null hypothesis indicates that the underlying mechanisms acting on species assemblages may reflect species interaction, abiotic filtering or dispersal limitation. To assess the co-occurrence patterns, we used the C-score index which quantifies the average number of checkerboard units calculated for each species pairs (Stone & Roberts 1990), and measures the degree of segregation across sampling sites. The co-occurrence null model randomizes the occurrence matrix based on different kinds of constraints (algorithms). In this study, the fixed-fixed (FF) algorithm was selected because it has a good Type I error rate, and is powerful at detecting patterns in noisy datasets, particularly when used with the C-score (Gotelli & Graves 1996; Gotelli 2000). With the FF-algorithm, species occurrence totals (rows) and species richness in each site (columns) are preserved. In other words, it retains differences among species in the number of sites they occupy (row sums) as well as among sites in the number of species they harbour (column sums) (Ulrich & Gotelli 2012). The C-score index was calculated for the empirical matrix and then compared statistically ($p < 0.001$) to the average of the C-score values calculated by randomization (n=1000) of each calculated matrix. For example, if the observed C-score is higher than the C-score average of the 1000 simulated matrices, then there is much less co-occurrence (segregation) in the observed matrix than expected by chance ($p < 0.001$). On the contrary, the observed matriw will show aggragated pattern, if observed C-score is lower than C-score average from simulated matrices.

References

Bougeard, S., Qannari, E.M. & Rose, N. (2011) Multiblock redundancy analysis: interpretation tools and application in epidemiology. *Journal of Chemometrics*, **25**, 467-475.

Carrascal, L.M., Galván, I. & Gordo, O. (2009) Partial least squares regression as an alternative to current regression methods used in ecology. *Oikos*, **118**, 681-690.

Gotelli, N.J. (2000) Null model analysis of species co-occurrence patterns. *Ecology*, **81**, 2606-2621.

Gotelli, N.J. & Graves, G.R. (1996) *Null models in ecology*. Washington, DC.

Stone, L. & Roberts, A. (1990) The checkerboard score and species distributions. *Oecologia*, **85**, 74-79.

Ulrich, W. & Gotelli, N.J. (2012) A null model algorithm for presence–absence matrices based on proportional resampling. *Ecological Modelling*, **244**, 20-27.
