# Supplementary material for: Factors shaping community assemblages and species co‐occurrence of different trophic levels
Source: Ecol Evol. 2017 May 23;7(13):4745–54. doi: 10.1002/ece3.3061 (PMC5496552; doi:10.1002/ece3.3061)
Supplement: Supplementary file 4 [file ECE3-7-4745-s004.pdf]

Appendix S4.

Multiblock Redundancy Analysis results- Importance of the five first dimensions *h* and associated cumulated percentages of variance of the datasets explained by the global component $t^{(h)}$ of mbRDA for plant ($\mathbf{Y}_{plant}$) and leafhopper ($\mathbf{Y}_{leafhopper}$) communities. $\mathbf{X}_1$: management; $\mathbf{X}_2$: topography; $\mathbf{X}_3$: chemical and physical property of soil; $\mathbf{X}_4$: structure of ground floor vegetation; $\mathbf{X}_5$: landscape composition defined within a 500-m radius; $\mathbf{X}_6$: landscape composition defined within a 200-m radius around the investigated vineyard; $\mathbf{X}_7$: first two PLRS components from the Partial Least-Squares Regression analysis.

$\mathbf{Y}_{plant}$ = Plant community	(*h* = 1)	(*h* = 2)	(*h* = 3)	(*h* = 4)	(*h* = 5)
% of inertia$^{(h)}$	23.4	8.7	5.8	5.5	3.8
Cum % of inertia	23.4	32.1	37.9	43.5	47.2
Cum % of variance of:					
**Y** expl. by $t^{(1-h)}$	19.7	30.2	37.6	46.5	51.8
**X** expl. by $t^{(1-h)}$	27.2	41.0	50.4	57.5	63.9
$\mathbf{X}_1$ expl. by $t^{(1-h)}$	16.2	35.2	42.0	56.3	60.2
$\mathbf{X}_2$ expl. by $t^{(1-h)}$	26.9	41.6	53.5	59.2	62.1
$\mathbf{X}_3$ expl. by $t^{(1-h)}$	27.7	34.4	43.5	58.4	63.8
$\mathbf{X}_4$ expl. by $t^{(1-h)}$	9.0	13.3	24.8	34.2	48.2
$\mathbf{X}_5$ expl. by $t^{(1-h)}$	23.8	36.2	40.5	42.0	54.5
$\mathbf{X}_6$ expl. by $t^{(1-h)}$	44.2	46.9	65.1	65.1	70.5
$\mathbf{X}_7$ expl. by $t^{(1-h)}$	42.7	79.3	83.7	87.2	88.1

$Y_{leafhopper}$ = Leafhopper community	(*h* = 1)	(*h* = 2)	(*h* = 3)	(*h* = 4)	(*h* = 5)
% of inertia$^{(h)}$	22.9	12.0	5.8	5.7	4.5
Cum % of inertia	22.9	34.9	40.7	46.3	50.9
Cum % of variance of:					
**Y** expl. by $t^{(1-h)}$	19.0	32.5	39.8	48.5	54.1
**X** expl. by $t^{(1-h)}$	24.8	40.1	49.3	57.7	64.4
$\mathbf{X}_1$ expl. by $t^{(1-h)}$	17.0	33.9	38.6	48.4	54.4
$\mathbf{X}_2$ expl. by $t^{(1-h)}$	24.1	36.2	44.7	57.7	60.4
$\mathbf{X}_3$ expl. by $t^{(1-h)}$	27.0	37.7	40.9	56.4	57.5
$\mathbf{X}_4$ expl. by $t^{(1-h)}$	8.1	9.8	35.0	43.2	70.2
$\mathbf{X}_5$ expl. by $t^{(1-h)}$	30.4	42.3	49.3	57.8	61.2
$\mathbf{X}_6$ expl. by $t^{(1-h)}$	29.5	47.9	56.8	59.7	62.8
$\mathbf{X}_7$ expl. by $t^{(1-h)}$	37.3	72.8	80.1	80.3	84.2
