# Supplementary material for: Factors shaping community assemblages and species co‐occurrence of different trophic levels
Source: Ecol Evol. 2017 May 23;7(13):4745–54. doi: 10.1002/ece3.3061 (PMC5496552; doi:10.1002/ece3.3061)
Supplement: Supplementary file 5 [file ECE3-7-4745-s005.pdf]

Appendix S5.

Significant plant and leafhopper species pairs selected from the G-C sub-matrix (62 Generalist leafhoppers and the 88 most widespread and abundant plants in the study sites) by Mean Bayes criterion in pairs co-occurrence analyses. Pairs of species are ordered by type of species pairs (**Pair**): l-l leafhopper-leafhopper; l-p leafhopper-plant; p-p plant-plant. **Occ1** and **Occ2:** occurrence of Species 1 and Species 2, respectively.

Pair	Species 1	Occ1	Species 2	Occ2	Joint Occ	P-value	Pattern
l-l	*Hyalestes obsoletus*	43	*Scaphoideus titanus*	38	26	3.22E-06	Segregated
l-l	*Megophtalmus scanicus*	43	*Scaphoideus titanus*	38	25	0.0007	Segregated
l-l	*Philaenus spumarius*	39	*Scaphoideus titanus*	38	25	0.0002	Segregated
l-l	*Deltocephalus pulicaris*	37	*Scaphoideus titanus*	38	24	1.43E-06	Segregated
l-l	*Deltocephalus pulicaris*	37	*Reptalus cuspidatus*	37	17	0.0000	Segregated
l-p	*Macrosteles cristatus*	50	*Plantago lanceolata*	36	26	0.0008	Segregated
l-p	*Zyginidia rhamni*	43	*Poa pratensis*	41	26	0.0034	Segregated
l-p	*Zyginidia rhamni*	43	*Plantago lanceolata*	36	24	1.8E-07	Segregated
l-p	*Hyalestes obsoletus*	43	*Plantago lanceolata*	36	25	1.01E-06	Segregated
l-p	*Hyalestes obsoletus*	43	*Holcus lanatus*	37	22	0.0000	Segregated
l-p	*Megophtalmus scanicus*	43	*Achillea millefolium*	38	25	0.0002	Segregated
l-p	*Megophtalmus scanicus*	43	*Holcus lanatus*	37	25	0.0002	Segregated
l-p	*Scaphoideus titanus*	38	*Holcus lanatus*	37	21	0.0000	Segregated
l-p	*Scaphoideus titanus*	38	*Rumex acetosa*	41	25	4.2E-07	Segregated
l-p	*Scaphoideus titanus*	38	*Stellaria media*	41	25	1.07E-05	Segregated
l-p	*Scaphoideus titanus*	38	*Arrhenatherum elatius*	41	22	0.0000	Segregated
l-p	*Philaenus spumarius*	39	*Holcus lanatus*	37	19	0.0000	Segregated
l-p	*Philaenus spumarius*	39	*Poa pratensis*	41	25	0.0017	Segregated
l-p	*Reptalus cuspidatus*	37	*Plantago lanceolata*	36	21	0.0000	Segregated
l-p	*Reptalus cuspidatus*	37	*Rumex acetosa*	41	25	0.0006	Segregated
l-p	*Reptalus cuspidatus*	37	*Veronica persica*	43	22	0.0000	Segregated
l-p	*Reptalus cuspidatus*	37	*Cerastium fontanum*	38	21	0.0000	Segregated
l-p	*Reptalus cuspidatus*	37	*Veronica arvensis*	38	15	0.0000	Segregated
l-p	*Deltocephalus pulicaris*	37	*Convolvulus arvensis*	42	25	0.0007	Segregated
l-p	*Deltocephalus pulicaris*	37	*Poa pratensis*	41	24	5.48E-06	Segregated
l-p	*Deltocephalus pulicaris*	37	*Cerastium fontanum*	38	23	0.0000	Segregated
l-p	*Deltocephalus pulicaris*	37	*Holcus lanatus*	37	16	0.0000	Segregated
l-p	*Deltocephalus pulicaris*	37	*Arrhenatherumelatius*	41	14	0.0000	Segregated
p-p	*Digitaria sanguinalis*	47	*Rumex acetosa*	41	27	0.0021	Segregated
p-p	*Lolium perenne*	45	*Veronica arvensis*	38	26	0.0011	Segregated
p-p	*Potentilla reptans*	45	*Holcus lanatus*	37	25	6.01E-05	Segregated
p-p	*Potentilla reptans*	45	*Plantago lanceolata*	36	22	0.0000	Segregated
p-p	*Veronica persica*	43	*Holcus lanatus*	37	18	0.0000	Segregated

Pair	Species 1	Occ1	Species 2	Occ2	Joint Occ	P-value	Pattern
p-p	*Convolvulus arvensis*	42	*Rumex acetosa*	41	26	0.0011	Segregated
p-p	*Convolvulus arvensis*	42	*Arrhenatherumelatius*	41	26	0.0004	Segregated
p-p	*Stellaria media*	41	*Cerastium fontanum*	38	25	1.42E-05	Segregated
p-p	*Poa pratensis*	41	*Plantago lanceolata*	36	25	0.0001	Segregated
p-p	*Rumex acetosa*	41	*Veronica arvensis*	38	22	0.0000	Segregated
p-p	*Cerastium fontanum*	38	*Holcus lanatus*	37	19	0.0000	Segregated
p-p	*Holcus lanatus*	37	*Plantago lanceola*	36	24	0.0000	Segregated
l-l	*Laodelphax striatella*	68	*Zyginidia pullula*	67	67	1.85E-06	Agregated
l-l	*Laodelphax striatella*	68	*Emelyanoviana mollicula*	66	66	6.00E-07	Agregated
l-l	*Laodelphax striatella*	68	*Psammotettix confinis*	64	64	1.71E-05	Agregated
l-l	*Laodelphax striatella*	68	*Anaceratagallia ribauti*	64	64	2.68E-05	Agregated
l-l	*Laodelphax striatella*	68	*Euscelis incisus*	63	63	3.73E-05	Agregated
l-l	*Laodelphax striatella*	68	*Arocephalus longiceps*	61	61	5.96E-06	Agregated
l-l	*Laodelphax striatella*	68	*Aphrodes makarovi*	53	53	0.0019	Agregated
l-l	*Laodelphax striatella*	68	*Empoasca pteridis*	52	52	0.0030	Agregated
l-l	*Laodelphax striatella*	68	*Cicadella viridis*	51	51	0.0017	Agregated
l-l	*Laodelphax striatella*	68	*Empoasca vitis*	51	51	0.0012	Agregated
l-l	*Laodelphax striatella*	68	*Balclutha punctata*	51	51	0.0028	Agregated
l-l	*Laodelphax striatella*	68	*Macrosteles laevis*	50	50	0.0045	Agregated
l-l	*Zyginidia pullula*	67	*Cicadella viridis*	51	51	0.0017	Agregated
l-l	*Zyginidia pullula*	67	*Balclutha punctata*	51	51	0.0008	Agregated
l-p	*Laodelphax striatella*	68	*Trifolium repens*	64	64	5.48E-06	Agregated
l-p	*Laodelphax striatella*	68	*Taraxacum officinalis*	62	62	0.0000	Agregated
