# Supplementary material for: Factors shaping community assemblages and species co‐occurrence of different trophic levels
Source: Ecol Evol. 2017 May 23;7(13):4745–54. doi: 10.1002/ece3.3061 (PMC5496552; doi:10.1002/ece3.3061)
Supplement: Supplementary file 6 [file ECE3-7-4745-s006.pdf]

Appendix S6.

Significant plant and leafhopper species pairs selected from S-H sub-matrix (56 Specialist leafhoppers and 34 potential Host plants) by Bayes M criterion in pairs co-occurrence analyses. Pairs of species are ordered by type of species pairs (**Pair**): l-l leafhopper-leafhopper; l-p leafhopper-plant; p-p plant-plAnthoxanthus **Occ1** and **Occ2:** occurrence of Species 1 and Species 2, respectively. (1) Data after Nickel & Remane 2002; in bold species did not recorded in vineyards of southern Switzerland.

**Pair**	**Species 1**	**Occ 1**	**Species 2**	**Occ 2**	**Join Occ**	**P-value**	**Plant host data from literature [(1)]**
l-l	*Adarrus exornatus*	33	*Ribautodelphax pungens*	12	11	0.0128	-
l-l	*Adarrus exornatus*	33	*Ditropsis flavipes*	9	8	0.0556	-
l-l	*Muelllerianella fairmairei*	17	*Eupelix cuspidata*	7	6	0.0030	-
l-l	*Megadelphax sordidula*	19	*Goniagnathus brevis*	5	4	0.0199	-
l-l	*Stenocranus major*	13	*Rhopalopyx elongata*	5	4	0.0070	-
l-l	*Ribautodelphax pungens*	12	*Rhopalopyx elongata*	5	4	0.0030	-
l-l	*Ebarrius cognatus*	9	*Eupelix cuspidata*	7	5	0.0004	-
l-l	*Ebarrius cognatus*	9	*Ribautodelphax collina*	4	3	0.0013	-
l-l	*Acanthodelphax spinosa*	4	*Acanthodelphax denticauda*	3	2	0.0000	-
l-p	*Adarrus exornatus*	33	*Carex caryophyllea*	14	12	0.0159	*Brachypodium pinnatum*
l-p	*Ribautodelphax pungens*	12	*Holcus lanatus*	36	10	0.0532	*Brachypodium pinnatum*
l-p	*Horvathianella palliceps*	2	*Chrysopogon gryllus*	1	1	0.0000	*Chrysopogon gryllus*
l-p	*Kelisia praecox*	1	*Chrysopogon gryllus*	1	1	0.0000	***Carex brizoides***
l-p	*Xanthodelphax straminea*	12	*Poa pratensis*	40	11	0.0267	*Agrostis capillaris*
l-p	*Muelllerianella fairmairei*	17	*Anthoxanthum odoratum*	32	14	0.0130	*Holcus mollis*
l-p	*Ebarrius cognatus*	9	*Anthoxanthum odoratum*	32	8	0.0086	*Festuca* spp.
l-p	*Ebarrius cognatus*	9	*Carex hirta*	18	7	0.0078	*Festuca* spp.
l-p	*Ebarrius cognatus*	9	*Carex caryophyllea*	14	7	0.0048	*Festuca* spp.
l-p	*Ebarrius cognatus*	9	*Thymus pulegioides*	14	6	0.0052	*Festuca* spp.
l-p	*Kelisia guttulifera*	10	*Carex hirta*	18	8	0.0035	*Carex sylvatica*
l-p	*Eupelix cuspidata*	7	*Carex caryophyllea*	14	6	0.0012	*Festuca rubra*
l-p	*Anakelisia perspicillata*	5	*Brachypodium pinnatum*	20	4	0.0271	***Carex flacca***
l-p	*Macrosteles lividus*	1	*Agrimonia eupatoria*	1	1	0.0000	***Eleocharis palustris***
l-p	*Macrosteles lividus*	1	*Holcus mollis*	1	1	0.0000	***Eleocharis palustris***
p-p	*Holcus lanatus*	36	*Leonthodon hispidus*	19	17	0.0060	-
p-p	*Anthoxanthus odoratum*	32	*Brachypodium pinnatum*	20	16	0.0042	-
p-p	*Anthoxanthus odoratum*	32	*Thymus pulegioides*	14	11	0.0299	-
p-p	*Leontodon hispidus*	19	*Carex caryophyllea*	14	10	0.0046	-
p-p	*Bromus erectus*	3	*Chrysopogon gryllus*	1	1	0.0000	-
p-p	*Agrimonia eupatoria*	1	*Holcus mollis*	1	1	0.0000	-
p-p	*Festuca arundinacea*	1	*Festuca rubra*	1	1	0.0000	-
