# Supplementary material for: Factors shaping community assemblages and species co‐occurrence of different trophic levels
Source: Ecol Evol. 2017 May 23;7(13):4745–54. doi: 10.1002/ece3.3061 (PMC5496552; doi:10.1002/ece3.3061)
Supplement: Supplementary file 7 [file ECE3-7-4745-s007.pdf]

Appendix S7.
Overview of results of the 3-steps statistical framework applied to plant and leafhopper communities.

	Type of Output		Description	Plant	Leafhopper	
**Step 1**	mbRDA optimal model		% variance explained	51.8	54.1	
	Block importance	Topography	% variance explained	19.9	24.6	
		Biotic	% variance explained	24.6	20.7	
	Variable importance	1° PLSR comp	% variance explained	21.6	11.4	
		Slope	% variance explained	14.7	18.5	
		Open area-500	% variance explained	11.6	n.s.	
**Step 2**	Variation partitioning	Abiotic	% variance explained	9.6	14.8	
		Biotic	% variance explained	4.9	3.8	
		Shared	% variance explained	12.5	20.5	
**Step 3**	Matrix-level approach (1)			(S)	(S)	
				**p-p**	**l-l**	**p-l**
	Pairwise approach	G-C matrix	n° significant associations	12 (S)	5 (S); 14 (A)	23 (S) 2 (A)
		S-H matrix	n° significant associations	7 (A)	9 (A)	15 (A)

(1) G-C matrix= Generalist leafhoppers-Common plant species matrix; S-H matrix= Specialist leafhoppers-potential Host plants matrix; S= segregation; A= aggregation; p-p= plant-plant; l-l= leafhopper-leafhopper; p-l= plant-leafhopper.
